# The Parental Overprotection Scale: Associations with child and parental anxiety^☆^

**DOI:** 10.1016/j.jad.2013.07.007

**Published:** 2013-11

**Authors:** Kiri Clarke, Peter Cooper, Cathy Creswell

**Affiliations:** a School of Psychology and Clinical Language Sciences, University of Reading, Whiteknights, Reading RG6 6AL, UK; b Psychology Department, Stellenbosch University, South Africa

**Keywords:** Child anxiety, Parent anxiety, Overprotection, Self-report

## Abstract

**Background:**

Parental overprotection has commonly been implicated in the development and maintenance of childhood anxiety disorders. Overprotection has been assessed using questionnaire and observational methods interchangeably; however, the extent to which these methods access the same construct has received little attention. Edwards et al. (2008, 2010) developed a promising parent-report measure of overprotection (OP) and reported that, with parents of pre-school children, the measure correlated with observational assessments and predicted changes in child anxiety symptoms. We aimed to validate the use of the OP measure with mothers of children in middle childhood, and examine its association with child and parental anxiety.

**Methods:**

Mothers of 90 children (60 clinically anxious, 30 non-anxious) aged 7–12 years completed the measure and engaged in a series of mildly stressful tasks with their child.

**Results:**

The internal reliability of the measure was good and scores correlated significantly with observations of maternal overprotection in a challenging puzzle task. Contrary to expectations, OP was not significantly associated with child anxiety status or symptoms, but was significantly associated with maternal anxiety symptoms.

**Limitations:**

Participants were predominantly from affluent social groups and of non-minority status. Overprotection is a broad construct, the use of specific sub-dimensions of behavioural constructs may be preferable.

**Conclusions:**

The findings support the use of the OP measure to assess parental overprotection among 7–12 year-old children; however, they suggest that parental responses may be more closely related to the degree of parental rather than child anxiety.

## 1 Introduction

Models of the development and maintenance of childhood anxiety disorders have commonly highlighted the central role of parental behaviours characterised by control and overprotection (Chorpita and Barlow, 1998; Murray et al., 2009; Rapee, 1997). These models have suggested that overprotective behaviours (in contrast to autonomy promotion) may convey to the child a sense that the world is dangerous, reinforce avoidance, and limit the child’s opportunities to develop skills and confidence in managing potential challenges. Indeed, parental overprotection (and specifically a lack of autonomy granting) is the parenting dimension most consistently associated with child anxiety symptoms and disorders (e.g. McLeod et al., 2007; Van der Bruggen et al., 2008; Wood et al., 2003).

Parental overprotection has typically been assessed on the basis of parent or child reports on questionnaire measures, or by independent ratings of observed parent-child interactions. It has been suggested that questionnaire based methods may underestimate associations with child anxiety (McLeod et al., 2007), for example, due to social desirability effects. The extent to which these different methods of assessing parental overprotection tap into the same behavioural constructs has received little attention (Van der Bruggen et al., 2008; although there is some evidence of modest associations between parent responses to parenting vignettes and observational assessments; McShane and Hastings, 2009), and the validity of conclusions based on parenting questionnaires is unclear (McLeod et al., 2007). One questionnaire measure which has received sound empirical scrutiny is the Parental Overprotection (OP) measure. This is a 19 item parent report questionnaire developed by Edwards et al. (2008, 2010) to assess parenting behaviours that restrict a child’s exposure to situations which the parent may perceive as being potentially threatening or harmful to the child. Notably, the responses given by parents of 3–5 year old children to this measure showed high levels of internal consistency, high levels of stability over time, and were found to correlate significantly with observer ratings of parental overprotective behaviours with their child in a physical threat task. Furthermore, both mothers' and fathers' scores on the OP measure were positively associated with change in child anxiety symptoms over a 12-month period (Edwards et al., 2010), suggesting that the measure also has good predictive validity.

The OP measure, therefore, is a potentially useful and efficient tool for the assessment of overprotective parenting behaviours; however, to date its use has been limited to preschool children from a community population. Given that the items included in the measure are likely to be applicable to older children (e.g. typical items include “I protect my child from his/her fears” and “I shelter my child from life's difficulties”), we were keen to examine its use in this context. We were also concerned to establish the utility of the measure in a clinical population. As studies of parenting in clinically anxious populations typically include children from about 7 years of age (Hudson and Rapee, 2001; Moore et al., 2004), our first aim was to evaluate the psychometric characteristics of the measure when completed by parents of 7–12 year old children.

Second, we set out to examine the association between OP scores and independent observations of parental behaviours; here we were interested to establish whether OP scores were specifically associated with observations of overprotective behaviours, and not other parental behaviours that have been found to be associated with child anxiety (i.e., those characterised by expressed anxiety or lack of positivity; McLeod et al., 2007; Wood et al., 2003).

Third, we investigated whether OP scores discriminate between children with a current anxiety disorder and non-anxious children, and whether associations were specific to anxiety, and were not accounted for by common comorbid difficulties (depression and conduct problems). Also, in view of Edwards et al.'s (2010) report of significant associations between OP scores and parental negative affect (anxiety, depression and stress), we were concerned to examine the extent to which the association between OP scores and child anxiety was accounted for by overlapping associations between child anxiety and parental anxiety or depression. We therefore set out to examine the association between OP and child anxiety, taking into account parental anxiety and depression. Finally, in previous reports, cross-sectional and longitudinal associations between OP and child anxiety were based entirely on parent reported child anxiety, which is known to be influenced by parental emotional states (Bernstein et al., 2005; Kroes et al., 2003; Lagattuta et al., 2012); we therefore examined the relative association between OP and child anxiety as reported by both parent and child.

## 2 Method

### 2.1 Participants

A sample of 90 children (51 male and 39 female), aged 7–12 years (*M*=9.3, SD=1.4), and their primary caregiving mothers, took part in the study. Of these, 60 children met diagnostic criteria for a primary diagnosis of an anxiety disorder (see below) and 30 children formed a non-anxious comparison group. This sample provided sufficient power (0.95) to detect a medium effect size (based on Edwards et al. (2008)) using multiple regression with up to four predictors.

Children in the anxiety disorder group were recruited through referrals to the Berkshire Child Anxiety Clinic at the University of Reading by local health and education service personnel. Children were only included if they had a primary diagnosis of an anxiety disorder based on a structured diagnostic interview with children and mothers (ADIS-C/P; see below). Primary anxiety disorders of the group were as follows: Separation Anxiety Disorder (26.7%), Generalised Anxiety Disorder (26.7%), Social Phobia (20%), Specific Phobia (16.7%), Agoraphobia without Panic Disorder (5%), and Anxiety Disorder Not Otherwise Specified (5%). The mean number of anxiety disorder diagnoses was 2.6 (SD=1.2) and the mean number of any diagnoses was 3.1 (SD=1.6).

Control participants were volunteers, recruited through invitation letters, sent predominantly through local schools and after school clubs, specifically asking for children to form a non-anxious comparison group. Children were screened on the basis of child and mother report on the Spence Children's Anxiety Scale- child and parent versions (SCAS-C/P; see below). Where children scored above the normal range (i.e., in the ‘borderline’ or ‘abnormal’ categories) they were thanked and not invited for further inclusion in the study.

Inclusion criteria across both groups required that both children and mothers did not have a known significant intellectual impairment, Autistic Spectrum Disorder (ASD) (determined by being registered within local learning disability and social communication services) or severe major depressive disorder, psychosis, or substance/alcohol dependence. Primary caregiver mothers could be either biological or adoptive mothers.

As shown in Table 1, the anxious and non-anxious groups did not differ according to ethnicity, socio-economic status, child age or gender. As planned, the two groups did differ on child anxiety symptoms (SCAS-P/C), and, as expected, they also differed on low mood (SMFQ-C) and conduct problems (SDQ-P). The groups did not, however, differ significantly on maternal self-reported anxiety (DASS-A) or depression (DASS-D). The majority of children (89%) came from families of White British origin, and from families whose socio-economic status was classified as higher/professional (74%; National Statistics Socio-Economic Classification, NS-SEC; HMSO, 2005).

### 2.2 Measures

#### 2.2.1 Parental Overprotection Measure

The parental overprotection measure (OP; Edwards et al., 2008) was used to measure mother self-reported overprotective behaviour. The OP consists of 19 items designed to assess parenting behaviours that restrict a child's exposure to perceived threat or harm, with items mainly having a behavioural or situation specific focus, rather than more general attitudes and beliefs (e.g. “When playing in the park I keep my child within a close distance of me” and “I protect my child from criticism”). Parents are asked to rate the extent to which the item represents their typical response of a 5 point scale ranging from 0 (not at all) to 4 (very much). The OP measure has previously been found to have high internal consistency (Cronbach's alpha=0.87), strong test–re-test reliability, and good construct and predictive validity when used with a community sample of parents of 3–5 year olds (Edwards et al., 2008).

For the current sample, the Cronbach's alpha of the OP scale was 0.89, indicating a high level of internal consistency. A similar internal consistency was found when looking at the anxious and control groups separately (*α*=0.90 and *α*=0.86 respectively). Frequency of responses across the five descriptors for each item was generally good, although for three items in the scale (“I would not allow my child to go out with family friends if I were not present”, “I am reluctant for my child to play some sports for fear he/she might get hurt”, and “I accompany my child on all outings”), ‘never’ was endorsed by the great majority of mothers (i.e., 80% or more). However, analyses indicated that the internal consistency of the scale would not be improved by the deletion of any items in the scale (for both the full sample and the anxious and control samples separately).

#### 2.2.2 Anxiety Disorders Interview Schedule for DSM IV for Children: Child and parent versions

The anxiety disorders interview schedule for DSM IV for children: child and parent versions (ADIS-C/P; Silverman and Albano, 1996), a structured diagnostic interview with well-established psychometric properties (Silverman et al., 2001), was used to assign children diagnoses. Where children met symptom criteria for a diagnosis (based on either child or mother report) they were assigned a clinical severity rating (CSR) ranging from 0 (complete absence of psychopathology) to 8 (severe psychopathology). As is conventional, only those children who met symptom criteria with a CSR of 4 or more (moderate psychopathology) were considered to meet diagnostic criteria, and when child and parent CSR scores were not in agreement, the highest CSR from either informant was used. Assessors (psychology graduates) were trained on the standard administration and scoring of the ADIS-C/P through verbal instruction, listening to assessment audio-recordings and participating in diagnostic consensus discussions. The first 20 ADIS-child and ADIS-parent interviews conducted were then discussed with a consensus team, led by an experienced diagnostician (Consultant Clinical Psychologist). The assessor and the consensus team independently allocated diagnoses and CSRs. Following the administration of 20 child and 20 mother interviews, inter-rater reliability for each assessor was checked, and if assessors achieved reliability of at least 0.85 they were then required to discuss one in six interviews with the consensus team to prevent inter-rater drift. Overall reliability for the team was excellent. Reliability for presence or absence of diagnosis was kappa=0.98 for both mother and child report; and for the CSR intra-class correlation was 0.97 for mother report and 0.98 for child report.

#### 2.2.3 Spence Children's Anxiety Scale

The Spence Children's Anxiety Scale (SCAS-C/P) was administered to assess child and mother reported symptoms of anxiety. Both child and parent versions ask the informant to rate how often the child experiences each of 38 anxiety symptoms. The child version also contains 6 positive filler items designed to reduce negative response bias. Both versions have demonstrated good concurrent validity and internal consistency (Spence, 1998; Nuata et al., 2004). Internal consistency based on data from the current sample was good (Cronbach's alpha=0.88 for SCAS-C and 0.95 for SCAS-P).

#### 2.2.4 Depression Anxiety Stress Scale

The Depression Anxiety Stress Scale (DASS-21; Lovibond and Lovibond, 1995) was used to assess both maternal anxiety and depression. The DASS-21 is a 21 item self-report measure of negative emotional states in adults which is comprised of three seven-item scales measuring depression, anxiety and stress. The DASS-21 is known to have good psychometric properties, with good internal consistency for all subscales (Antony et al., 1998). Internal consistency based on data from the current sample was good (Cronbach's alpha=0.89 for the depression subscale and 0.78 for the anxiety subscale).

#### 2.2.5 Short Mood and Feelings Questionnaire

The Short Mood and Feelings Questionnaire (SMFQ; Angold et al., 1995) was administered to assess child reported symptoms of low mood. The SMFQ-C is a brief, 13 item measure which requires children to report how often in the previous 2 weeks they had experienced a number of symptoms. The SMFQ-C has demonstrated high concurrent validity and good internal reliability (Angold et al., 1995). Internal consistency based on data from the current sample was good (Cronbach's alpha=0.86).

#### 2.2.6 Strengths and Difficulties Scale: Conduct subscale

The Strengths and Difficulties Scale: conduct subscale (SDQ; Goodman, 1997) was used to assess mother reported child behavioural disturbance. The SDQ is known to have good psychometric properties (Goodman, 1997, 2001). The parent report version of the SDQ was used as parents are often considered to be most reliable in terms of providing information on children's externalising symptoms (Grills and Ollendick, 2002; Edelbrock et al., 1985). Internal consistency based on data from the current sample was acceptable (Cronbach's alpha=0.66).

### 2.3 Procedure

Following referral to the Berkshire Child Anxiety Clinic, participants were invited for an initial assessment appointment in which the diagnostic interviews and symptom questionnaires were administered to mothers and children in separate rooms. Children were assisted in completing the questionnaires by a research assistant in case of literacy difficulties. Any child found to meet diagnostic criteria for a current anxiety disorder was invited to take part in a research assessment before beginning treatment at the clinic.

The research was approved by both the University of Reading Research Ethics Committee and the Berkshire Local Research Ethics Committee. Before taking part, both parents and children read study information sheets and signed forms to indicate that they understood what was involved, were willing to take part and that they were aware that they could withdraw from the study at any time. Participation in research assessments was voluntary, travel expenses were paid and decisions regarding whether to take part did not affect access to treatment.

For the control children, mothers were sent the SCAS-P and SDQ-P to complete and return. If mothers reported that children scored within normal limits on the SCAS-P, an appointment was scheduled for the research assessment. Control children completed the SCAS-C and SMFQ-C during the laboratory assessment with the help of a research assistant. Families in the control condition were given £25 gift tokens in exchange for taking part.

For both clinic and control participants, research assessments were carried out in a clinic laboratory fitted with CCTV style cameras. Assessments first involved a 5 min acclimatisation task (a ‘Connect Four’ game), followed by three challenge tasks which the mother and child completed together following the procedure of Creswell et al. (2012). The three tasks were designed to allow observations of maternal behaviours across a range of potential stressors, representing mild social, performance and physical threats.

#### 2.3.1 Speech task

For the first task the child was asked to prepare and give a 3–5 min speech. The mother and child were given 5 min alone to choose from a list of four topics and to prepare a speech on the topic. After this time the research assistant re-entered the room with a video camera and the mother was asked to introduce her child to the camera before taking a seat on a sofa at the side of the room whilst the child gave the speech. The mother was told before the task began that should her child require help, it was up to her to decide what was appropriate.

#### 2.3.2 Tangram task

The second task followed the procedure of Hudson and Rapee (2001). The child was given two difficult tangram puzzles to complete. He/she was told it was a test of thinking ability, and he/she had 5 min to complete the puzzles. The mother was given a sheet showing the puzzle solutions, but told to only use them if she felt her child really needed the help.

#### 2.3.3 Black box task

In the third task, the child was presented with a black box with a hole in each of the four sides, obscured by a black curtain. The child was told that there were four scary items in the black box and the child should try and find out what they were. The box contained four fluffy or squidgy toys (e.g. slime). The mother was told before the task began that she could help her child as much as she felt was necessary.

### 2.4 Maternal behavioural coding

The video recordings of the tasks were coded, by psychology graduates, using a scheme which was adapted, to be age appropriate, from the scheme developed by Murray et al. (2011). Maternal behaviours were rated on a scale of 1–5, where 1 represents no signs of that behaviour and 5 represents very strong signs of that behaviour. Behaviours were coded minute by minute, giving a score between 1 and 5 for each minute, and the mean score across all the minutes coded (for each dimension) was used for analysis. Before coding the material used for analysis in this study, inter-rater reliability was checked by carrying out mixed single measures intraclass correlations between the scores of the coders and a master coder for a set of 25 practice videos. Reliability was between 0.6 and 1, with a mean intraclass correlation of 0.84, for all raters across all dimensions.

The mother–child interactions used were coded according to a coding scheme which attempts to define parenting behaviours as specifically as possible, as previous research has highlighted the importance of disaggregating broad parenting dimensions, which can underestimate the strength of parenting–childhood anxiety associations (McLeod et al., 2007). Edwards et al. (2008) defined overprotection as a “style of parenting that is overly restrictive when it comes to protecting the child from potential harm or risk”. Such items refer to two related aspects of overprotection; (i) harm minimisation (i.e., going beyond what is required to protect the child's emotional wellbeing, reflected by items on the OP measure such as “I comfort my child immediately when he/she cries”), and (ii) intrusiveness (i.e., behaviours which restrict the child’s autonomy, which is reflected by items such as “I try to protect my child from making mistakes”). Although we would usually advocate the use of highly specific parenting dimensions (Murray et al., 2011; Creswell et al., 2012), in order to be consistent with what is being measured by the OP measure, we combined observations of these two sub-dimensions of overprotection to assess their relationship to the OP measure.

The maternal behaviours rated were as follows:

i. **Overprotection.** Parenting that is overly restrictive when it comes to protecting the child from potential harm or risk. This dimension consists of the combination of two specific sub-dimensions of overprotection:
  a. **Harm minimisation.** The degree to which the mother goes beyond what is required to protect her child, by comforting and showing concern about the emotional state of the child where it is not warranted (i.e., the child is showing little or no distress), such as remaining standing close to the child during the speech task when the child is not showing distress, or telling the child not to worry during the tasks when the child is not struggling or showing distress.
  b. **Intrusiveness.** The degree to which the mother restricts the child's autonomy or is inappropriately directive and controlling in her help. Includes both verbal intrusiveness, such as telling the child what to do and making decisions for the child (e.g. choosing the topic for the speech task or telling the child where to place puzzle pieces on the tangram task) and physical intrusiveness such as taking over the task from the child (e.g. placing pieces of the puzzle in the tangram task or taking objects out of the black box themselves).
ii. **Expressed anxiety.** How anxious the mother appears during the task, measured by taking face (e.g. facial twitches or grimaces), body (e.g. wringing of the hands and fidgeting) and speech (e.g. stumbling over words) signs into account.
iii. **Positive behaviour.** The general emotional climate the mother provides the child (warmth), including physical affection, expression of positive regard for the child and general demeanour, as well as the extent to which the mother positively motivates the child to complete the task (encouragement), including tone of voice and encouraging statements. Warmth and encouragement were coded separately; however, ratings correlated highly (*r*=0.70, *p*≤0.001), so these scales were combined.

## 3 Results

### 3.1 Preliminary analyses

Continuous data were screened in relation to the assumptions of parametric tests (Tabachnick and Fidell, 2000). The following variables were not normally distributed: SDQ-P conduct score, SMFQ-C total, overprotection for all three tasks, and maternal expressed anxiety for the tangram and black box task. Log transformations improved the distribution of these variables and were used in the primary analyses. SDQ-conduct score remained positively skewed and therefore the analyses involving this variable were repeated with both the parametric test using 1000 bootstrap samples as well as the non-parametric equivalent to ensure findings were robust. Results were consistent, so, for simplicity, the parametric test results are reported. Maternal expressed anxiety on the tangram task also remained highly positively skewed due to the generally low level of anxiety expressed by mothers in this task. This variable was therefore dichotomised to represent ‘no anxiety’ versus ‘some anxiety’.

Preliminary analyses were conducted to identify potential confounding variables. There was no significant association between OP score and child gender (*t*(88)=−0.69, *p*=0.49), socioeconomic status (high socioeconomic status versus other; *t*(21.07)=−0.73, *p*=0.48), ethnicity (White British versus other; *t*(88)=−1.256, *p*=0.21), low mood (SMFQ score; *r*=0.11, *p*=0.33), and behavioural disturbance (SDQ-conduct score; *r*=0.16, *p*=0.14). OP score was, however, significantly associated with child age in months (*r*=−0.22, *p*=0.04), with mothers of younger children tending to report higher overprotection. OP scores were also significantly associated with maternal depression (DASS-D; *r*=0.35, *p*=0.001), with mothers who scored higher on symptoms of depression tending to report higher overprotection. Subsequent analyses therefore controlled for child age (in months) and maternal depression scores, as appropriate.

### 3.2 Associations between OP and observational data

As shown in Table 2, the OP score did not correlate significantly with any of the maternal behavioural variables for the speech or black box tasks (*r*=−0.15 to 0.13). For the tangram task there was a significant association between the OP score and maternal overprotection (*r*=0.25, *p*=0.016), but not positive behaviours (*r*=0.04, *p*=0.69) or maternal expressed anxiety (*t*(25.83)=0.10, *p*=0.92). This suggests that the OP score is specifically related to observed overprotective maternal behaviours within specific contexts, here a difficult puzzle task.

### 3.3 Associations between OP and child and maternal anxiety

As shown in Table 1, the child anxiety group was not significantly associated with the OP score (mean (SD): anxious—26.19 (SD=13.10), nonanxious—26.55 (SD=11.04)). Group scores on observational measures of parental behaviours are shown in Table 3. Consistent with the OP self-report findings, observed overprotection was not significantly associated with child anxiety status in any of the three tasks, although expressed anxiety and positive behaviours were both higher among parents of children with anxiety disorders compared to parents of non-anxious children.

Multiple regression analyses in which the OP score was the dependent variable and child anxiety was entered as a continuous dependent variable, using either the SCAS-C or the SCAS-P, and controlling for child age, also showed the same pattern of non-significant results (SCAS-C: *β*=−0.11, *p*=0.27; SCAS-P: *β*=−0.08, *p*=0.45). The association between the OP score and maternal anxiety (symptoms, DASS-A) was also examined within a multiple regression analysis, controlling for child age and maternal depression. As shown in Table 4, maternal anxiety was significantly associated with OP (*β*=0.27, *p*=0.03), even after controlling for maternal depression.

## 4 Discussion

The first aim of this study was to assess the psychometric properties of the OP scale when used with parents of children aged 7–12 years, including those from a clinically anxious population. Like Edwards et al. (2008), we found that the OP score reduced with child age; so, as would be expected given the older age group we studied, the mean OP score reported in the current sample was somewhat lower than that reported by Edwards et al. (2008); (26.31 compared to 32.78). Nonetheless, despite being designed for use with younger children, the questionnaire items generally evoked a broad range of responses and the scale was found to have a high level of internal consistency.

We found that the OP score was significantly associated with observed overprotection in a challenging puzzle (Tangram) task. This is encouraging, as the tangram task has been frequently used in studies of parenting in the context of child anxiety disorders (Hudson and Rapee, 2001; Creswell et al., 2012). It is important to bear in mind that, while the magnitude of association was statistically significant and similar to that reported for overprotection by Edwards et al. (2008), it was in the small–medium range (*r*=0.25). Nevertheless, given the difference in methods used and the specific laboratory-based context of the observational task (compared to the more everyday scenarios referred to in the OP measure), it is striking that a significant association was found. It is interesting that parent reported OP was not significantly associated with overprotective behaviours as observed in the black box (physical threat) and speech (social threat) tasks. While this finding may suggest a lack of validity of the measure, it is consistent with the suggestion that the type of task used influences the degree to which parents express particular behaviours (Edwards et al., 2008; Hudson et al., 2008; Murray et al., 2011). It is notable that the significant association we did find between the OP score and observed parental behaviour was specific to the construct of parental overprotection; and no association was found between OP score and other parental responses that have previously been implicated in the development and maintenance of child anxiety (i.e., expressed anxiety and a lack of positivity).

Contrary to expectations, OP scores were not higher for children with a current anxiety disorder than for the comparison non-anxious children. This was unexpected as it has previously been found within a preschool community sample that OP was significantly associated with child anxiety symptoms, both cross-sectionally (Edwards et al., 2008) and longitudinally (Edwards et al., 2010). The lack of association between the OP score and child anxiety within our study, even when both child anxiety and overprotection were assessed by parental report (as in Edwards et al. (2010)) was unexpected. Consistent with Edwards et al. (2010), however, OP scores were significantly associated with parental anxiety symptoms, and this effect held even after controlling for parental depression. Both of these findings are, however, consistent with the findings of Schneider et al. (2009), who observed that mothers with panic disorder were more verbally controlling than non-anxious mothers during an interaction task with their child (mean age=17.7 years, SD=2.5 years), regardless of the anxiety status of the child, and this effect also held after controlling for parental depression. The discrepant results between both Schneider and the current study, on the one hand, and Edwards et al. (2010) on the other, might suggest that parental overprotection may be of particular importance in relation to the development of anxiety among younger (e.g. preschool) children.

The association between the OP score and parental anxiety suggests a potential implication for the treatment of child anxiety. It has previously been found that elevated parental anxiety can impede optimal treatment outcomes for children with anxiety disorder (Cobham et al., 1998). One possibility is that this association is driven by anxious parents responding to their child over-protectively; that is, in a manner that runs contrary to the purpose of treatment (i.e., to encourage children to challenge negative expectations and face fears; e.g. Creswell et al., 2012). It would therefore be valuable to investigate whether parental overprotection as measured by the OP scale is associated with treatment outcome. If it is, the OP measure may be a useful tool for the assessment of a parental style predictive of treatment outcome for children with anxiety disorders.

## 5 Limitations

The findings of the current study need to be interpreted with various limitations in mind. The participants were predominantly from a fairly affluent social group and of non-minority status. This limits the generalisability of the findings. However, our sample is similar to that of Edwards et al. (2008, 2010), who recruited participants of non-minority status from predominantly middle-to-high income households, so this is unlikely to explain the difference in results between the current study and that of Edwards et al. (2008, 2010). It should also be noted that all participating primary caregivers were mothers. While this largely reflects the caretaking roles within the local population, understanding the role of paternal behaviours in the course of child anxiety is, of course, equally important and must be addressed in future research. As noted above, given the different contexts of the observational tasks and the situations referred to in the OP measure, it is striking that a significant association was found between parenting as assessed by these different methods. The inclusion of observational assessments of more naturalistic situations in which children face potential challenges would allow for a more ecologically valid test of the association between these two methods of assessment. For this research we combined two specific sub-dimensions of overprotection: harm minimisation and intrusiveness. It was appropriate to combine these here as items in the OP measure reflect both of these types of behaviours. However, it has been suggested that the use of highly specific sub-dimensions of behavioural constructs is preferable (McLeod et al., 2007), and research which investigates the relationship between self-report and observed parenting which attempts to disaggregate the construct of overprotection further may be beneficial.

## 6 Strengths and conclusions

The strengths of the current study include that it is among the first to examine the association between parent report and observational assessments of parenting dimensions commonly found to be associated with child anxiety. In addition, we used clinician ratings of child anxiety, independent behavioural observations of parenting behaviours, and took account of parental anxiety and commonly comorbid difficulties. The current results support the use of the OP scale as a reliable and valid measure of overprotective parental behaviours with children in mid to late childhood; however, interestingly, within this age group, the measure was not found to be associated with the presence of level of child anxiety, but it was associated with the level of parental anxiety.

## Role of funding source

The research described was conducted as part of studies funded by the Medical Research Council, awarded to Cathy Creswell and Peter Cooper.

## Conflict of interest

All authors declare that they have no conflicts of interest.

## Figures and Tables

**Table 1:** Sample characteristics for demographics and self-report measures.

	Anxious group	Non anxious group	Chi-square
	*N* (%)		

White British (other)	50 (10)	25 (5)	*χ*^2^(1)=0
Higher professional (other)^a^	42 (14)	25 (4)	*χ*^2^(1)=1.44
Male (female)	34 (26)	17 (13)	*χ*^2^(1)=0

	Mean (SD)		*t*-test

Child age (in months)	116.03 (18.14)	116.67 (16.35)	*t*(88)=0.87
SCAS-C^b^	38.12 (14.64)	26.69 (10.25)‘	*t*(86)=3.77⁎⁎⁎
SCAS-P^b^	39.09 (17.24)	13.37 (5.09)	*t*(73.711)=10.51⁎⁎⁎
SMFQ-C^b^	7.32 (5.34)	4.17 (3.12)	*t*(83.301)=3.48⁎⁎
SDQ-P	2.60 (2.01)	1.27 (1.20)	*t*(85.071)=3.92⁎⁎⁎
DASS-D	6.97 (6.66)	5.07 (5.40)	*t*(88)=1.35
DASS-A	5.10 (5.99)	4.33 (3.97)	*t*(88)=0.63
OP score	26.88 (13.24)	26.97 (11.09)	*t*(88)=−0.03

*Note*: SCAS-C=Spence Child Anxiety Scale, child report; SCAS-P=Spence Child Anxiety Scale, parent report; SMFQ-C=Short Mood and Feelings Questionnaire, child report; SDQ-P=Strengths and Difficulties Questionnaire—conduct subscale, parent report; DASS-D=Depression Anxiety Stress Scales—depression subscale; DASS-A=Depression Anxiety Stress Scales—anxiety subscale; OP score=Parental Overprotection Measure score.

⁎⁎ *p*<0.01.

⁎⁎⁎ *p*<0.001.

a Five cases missing data and therefore not included in analysis.

b Two cases missing data and therefore not included in analysis

**Table 2:** Correlation coefficients (Pearson's *r*) between OP score and the observational behavioural ratings for each task.

	Speech	Tangram	Black box
Overprotection	0.05	0.25*	−0.13
Expressed anxiety	−0.05	n/a	−0.15
Positive behaviour	−0.07	0.04	0.13

⁎ *p*<0.05.

**Table 3:** Observations on behavioural tasks.

	Anxious group	Non anxious group	
	Mean (SD)		*t*-test
**Speech task**			
Overprotection	1.25 (0.31)	1.29 (0.26)	*t*(88)=0.57
Expressed anxiety	2.19 (0.63)	1.89 (0.60)	*t*(88)=−2.15⁎
Positive behaviour	2.84 (0.47)	2.59 (0.63)	*t*(88)=−2.1⁎

**Tangram task**			
Overprotection	1.36 (0.45)	1.37 (0.30)	*t*(88)=0.19
Expressed anxiety	1.11 (0.22)	1.06 (0.14)	*t*(82.54)=−1.35
Positive behaviour	2.78 (0.77)	2.58 (0.51)	*t*(81.28)=−1.47

**Black box task**			
Overprotection	1.49 (0.46)	1.53 (0.48)	*t*(88)=0.37
Expressed anxiety	1.76 (0.69)	2.05 (0.81)	*t*(88)=1.79
Positive behaviour	3.48 (0.82)	2.83 (0.91)	*t*(88)=−3.40⁎⁎

⁎ *p*<0.05.

⁎⁎ *p*<0.01.

**Table 4:** Association between maternal anxiety and OP score, controlling for child age and maternal depression.

	***B***	**SE**	***β***
Step 1			
Constant	41.98	8.24	
Child age in months	−0.17	0.07	−0.24*
Maternal depression	0.702	0.19	0.35***

Step 2			
Constant	40.15	8.12	
Child age in months	−0.16	0.07	−0.22*
Maternal depression	0.36	0.25	0.18
Maternal anxiety	0.63	0.29	0.27*

*Note*: *R*^2^=0.17 for Step 1, *∆R*^2^=0.22 for Step 2 (*p*=0.03).

⁎ *p*<0.05.

⁎⁎⁎ *p*<0.001.
